# Clinical significance and intestinal microbiota composition in immunocompromised children with norovirus gastroenteritis

**DOI:** 10.1371/journal.pone.0266876

**Published:** 2022-04-20

**Authors:** Pei-Chun Lin, Yu-Chen S. H. Yang, Sheng-Chieh Lin, Meng-Che Lu, Yin-Tai Tsai, Shou-Cheng Lu, Shu-Huey Chen, Shih-Yen Chen

**Affiliations:** 1 Division of Pediatric Gastroenterology, Department of Pediatrics, Shuang Ho Hospital, Taipei Medical University, Taipei, Taiwan; 2 Joint Biobank, Office of Human Research, Taipei Medical University, Taipei, Taiwan; 3 Division of Allergy, Asthma and Immunology, Department of Pediatrics, Shuang Ho Hospital, Taipei Medical University, Taipei, Taiwan; 4 Department of Pediatrics, School of Medicine, College of Medicine, Taipei Medical University, Taipei, Taiwan; 5 Department of Medicine Laboratory, Shuang Ho Hospital, Taipei Medical University, New Taipei City, Taiwan; 6 Division of Pediatric Hematology and Oncology, Department of Pediatrics, Shuang Ho Hospital, Taipei Medical University, Taipei, Taiwan; Universita degli Studi di Parma, ITALY

## Abstract

**Background:**

Norovirus (NoV) infection is common in pediatric patients with immunodeficiency and is more likely to cause severe disease. Objective Our study aims to figure out the clinical differences and distribution of intestinal microbiota in immunocompromised children with NoV gastroenteritis.

**Methods:**

Pediatric patients admitted to Shang-Ho Hospital with diagnosis of acute gastroenteritis including different immune status were enrolled and their medical records were reviewed. NoV gastroenteritis was validated using RT-PCR molecular methods. Viral shedding period was determined by real-time RT-PCR assays. Intestinal microbiota enrichment analysis was carried out by next generation sequencing after fecal DNA extraction and subsequent Linear Discriminant Analysis (LDA) Effect Size (LEfSe) method.

**Results:**

Significantly higher frequency of diarrhea [mean, (IQR), 3.8 (3–5) /day] and longer viral shedding time [mean, IQR, 8.5 (5–13) days] was found in immunocompromised NoV infections than in immunocompetent patients without NoV infections (p = 0.013*) and immunocompetent patients with NoV infections (p = 0.030**). The fever prevalence was significantly lower in immunocompromised NoV infections than in different immune or infection status. Intestinal microbiota metagenomics analysis showed no significant community richness difference while the LEfSe analysis showed a significant difference in commensal richness at the phylum level, the family level, and the genus level in patients under different immune status.

**Conclusion:**

We evaluated the clinical significances and microbiota composition in immunocompromised children with norovirus gastroenteritis. This will further facilitate studies of the interaction between the intestinal microbiota in such patients with precise determination of their bacterial infection control and probiotic supplements strategy.

## Introduction

Norwalk-like viruses or noroviruses (NoVs) replaced rotaviruses as the leading cause of viral acute gastroenteritis (AGE) in humans worldwide after the launch of a rotavirus vaccine [1, 2]. Molecular methods have revealed the detailed genetic and molecular features of circulating NoVs, although their rapid evolution and genetic diversity have made NoV identification, classification, surveillance, and vaccine development difficult [3, 4].

Over the past decade, our research has shown that NoV infections in Taiwan were caused by different epidemic strains associated with complications and uncommon clinical manifestations, increasing the clinical impact of NoV disease in Taiwan over time [5, 6]. NoV infection is common in immunodeficient pediatric patients, in whom it is more likely to cause severe disease with prolonged virus shedding, and even in-host evolution of the virus. Transmission is an important source of diversity at the inter-host level of NoV evolution, and chronically infected immunocompromised subjects are a potential reservoir for the emergence of new viral variants [7]. A study of NoV diversity in recurrent persistent diarrhea in immunocompromised patients provided data on within-host evolution in chronic NoV infections [8]. Recently, we found mutant recombinant genotype NoV strains with sustained high viral loads in pediatric patients [9]. Another study found that the bacterial microbiome prevented persistent murine norovirus (MNV) infection via the replenishment of the bacterial microbiota related to host immune specificity [10]. Therefore, this study examined clinical differences in the intestinal microbiota in immunocompromised patients with norovirus gastroenteritis.

## Materials and methods

### Study design, patients enrollment

This study enrolled pediatric patients (under 18 years of age) hospitalized in Shang-Ho Hospital with a diagnosis of acute gastroenteritis who presented diarrhea, vomiting, or fever accompanied, including immunocompetent and immunocompromised patients randomly irrespectively of gender, ethnicity, and hospitalization ward during August of 2019 to July of 2020. The study was approved by Taipei Medical University Joint Institutional Review Board (TMUJIRB) No. N201903046. After the written type informed consent was obtained from a legal guardian of each subject involved in the study, 2 mL stool samples were collected from the children in the ward after hospitalization within 3 days. All samples were stored at –70oC before extraction. All methods were carried out in accordance with relevant guidelines and regulations.

#### In-hospitalization clinical data

Clinical and demographic data were obtained from the patients’ medical records, including age, gender, symptoms (diarrhea frequency and duration, vomiting frequency and duration, dehydration status, fever, blood in stool, abdominal pain, and bilious vomiting) and laboratory findings. All data were fully anonymized before assessed and after the written type informed consent was obtained.

#### Nucleic acid extraction and reverse transcriptase polymerase chain reaction

Viral nucleic acids were extracted from the fecal samples using a QIAamp Viral RNA Mini kit (QIAGEN), according to the manufacturer’s recommendations. The concentration of viral nucleic acids was determined using a NanoDrop 1000 spectrophotometer (Thermo Fisher Scientific). cDNA synthesis and polymerase chain reaction (PCR) were performed according to the manufacturer’s recommendations (SuperScript III First-Strand Synthesis System; Invitrogen). The PCR primers and conditions used to determine norovirus genotypes were described previously [11]. NoV sequences were identified using the Norovirus typing tool website (RIVM) and uploaded to the National Center for Biotechnology Information (NCBI) database (http://www.ncbi.nlm.nih.gov).

#### Virus shedding analysis

Primers were designed for NoV RdRp gene sequencing. The reactions used 10-fold serial dilutions of norovirus GII.4 DNA as positive controls at starting concentrations of 10^8^ DNA copies/mL. To evaluate the amplification efficiency of the real-time RT-PCR assays, standard curves were generated for NoV GII.4 DNA copy numbers versus Cq values [12]. The coefficient of determination (R2) in the linear regression analysis was 0.99, indicating a strong correlation between the copy number and Cq value. The virus shedding period was the interval from the peak viral load to the virus becoming undetectable. Fisher’s exact test was used to evaluate the significance of differences in clinical features. The significance of differences between two independent samples was analyzed using the nonparametric Mann–Whitney U-test.

#### Intestinal microbiota metagenomics

Stool total DNA was extracted using the QIAamp Fast DNA Stool Mini Kit (QIAGEN, Hilden, Germany), according to the manufacturer’s recommendations. Universal primers (341F and 805R) for the 16S variable regions V3–4 were used for PCR amplification.

The purified amplicon mixtures were sequenced using the Illumina MiSeq System according to the manufacturer’s protocols (Illumina, San Diego, CA, USA). The 16S rRNA regions were amplified using KAPA HiFi HotStart ReadyMix (Roche, Manheim, Germany). The PCR amplicons were purified using Agencourt® AMPure® XP Reagent (Beckman Coulter, Brea, CA, USA) and quantified using an Agilent Bioanalyzer (Agilent Technologies, Santa Clara, CA, USA). The amplified V3–V4 regions of the bacterial 16S rRNA genes were first removed from the demultiplexed, paired reads using Cutadapt (v 1.12; doi: 10.14806/ej.17.1.200). The filtered reads were processed in the R environment (v 3.6.1) using R package DADA2 (v 1.14.1) [13], following the workflow described in Callahan et al. [14], without the rarefying procedure. Briefly, the forward and reversed reads were filtered and trimmed based on the read quality score and read length. Dereplication was then performed to merge identical reads, and the reads were subjected to the denoise DADA2 algorithm, which alternates between error-rate estimation and sample composition inference until they converge on a jointly consistent solution. Finally, the paired reads that required a minimal 20 bp overlap were merged and chimeras were removed. At this point, we obtained a list of V3–V4 sequence variants (SVs) in our samples that were inferred by DADA2, as well as the frequency of each SV in each sample. Taxonomy was assigned using the SILVA database (v128) as the reference [15], with a minimum bootstrap confidence of 80%. The SVs were aligned with DECIPHER (v2.14.0) and a phylogenetic tree was constructed using phangorn (v2.2.5) [16]. The count table, taxonomy assignment results, and phylogenetic tree were consolidated into a phyloseq object, and community analyses were performed using phyloseq (v1.30.0) [17]. The alpha-diversity indices were calculated using the estimate_richness function in the phyloseq package. The treatment and control were compared using the exact Wilcoxon–Mann–Whitney test (at α = 0.05). UniFrac distances were calculated using GUniFrac (v1.1) to assess the community dissimilarity between groups [18]. Principal coordinate analysis (PCoA) ordination on UniFrac distances was performed; the adonis and betadisper functions in the vegan package (v2.5.6; https://CRAN.R-project.org/package=vegan) were used to analyze the dissimilarity of composition among groups and the homogeneity of dispersion, respectively. Microbiota enrichment analysis between groups was done using the linear discriminant analysis (LDA) effect size (LEfSe) method with alpha set at 0.05 (Kruskal–Wallis and Wilcoxon tests) and a logarithmic LDA score ≥ 2 [19], and visualized as cladogram using GraPhlAn [20].

#### Statistical analysis

Continuous clinical data were analyzed using Student’s t-tests and expressed as means (interquartile range). Binary data were analyzed using the χ 2 test. A value of P < 0.05 was considered to indicate statistical significance. All tests were performed using SAS software v. 8 for Windows (SAS Institute Inc., Cary, NC, USA).

## Results

After excluding eight of the 66 subjects because of incomplete data, 58 stool samples were collected: 17 from pediatric patients with norovirus AGE (mean age, 68 [range 23–124] months) and 41 from non-norovirus AGE children (mean age, 56 [range 20–86] months). Of the 17 norovirus AGE patients, four were immunocompromised (three acute lymphoblastic leukemia and one lymphoepithelioma-like carcinoma of the lung) and 13 were immunocompetent. The 41 non-norovirus AGE children comprised seven immunocompromised (four acute lymphoblastic leukemia, one yolk sac tumor, one choroid plexus carcinoma, and one systemic lupus erythematosus) and 34 immunocompetent patients. All fecal samples were collected at least 1 week after discontinuing antibiotics.

### Clinical manifestations of acute gastroenteritis by immune status

Table 1 summarizes the clinical findings, including diarrhea frequency (times/day), vomiting frequency (times/day), prevalence of fever, and fever duration (days). Diarrhea was significantly (P = 0.013) more frequent in immunocompromised NoV infections [mean, interquartile range (IQR), 3.8 (3–5)] than in immunocompetent patients without NoV infection [mean, IQR, 2 (0–3)]. Vomiting was significantly more frequent in immunocompetent patients without NoV infections [mean, IQR, 1.8 (1–3)] than in immunocompromised patients with NoV infections [mean, IQR, 1 (0–1); P = 0.03] or those without NoV infections [mean, IQR, 0.9 (0–1); P = 0.015]. Fever was significantly less prevalent in immunocompromised patients with NoV infections (25%) than in immunocompetent patients with NoV infections (85%; P = 0.022) or immunocompromised patients without NoV infections (71%; P = 0.035). Fever duration was longest in immunocompetent patients with NoV infections [mean, IQR, 2 (1–2)], although the difference was not significant. Virus shedding time was significantly (P = 0.03) longer in immunocompromised patients with NoV infections [mean, IQR, 8.5 (5–13)] than in immunocompetent patients with NoV infections [5.9 (2.6–7.1)].

**Table 1:** Clinical significances of children with or without norovirus infection under different immunity characteristics.

Patients’ characteristics	Immunocompromised NoV infections (N = 4)	Immunocompetent NoV infections (N = 13)	Immunocompromised non-NoV infections (N = 7)	Immunocompetent non-NoV infections (N = 34)	*P* value
Diarrhea frequency (IQR), times/day	3.8* (3–5)	3.5 (3–5)	3.1 (3–4)	2* (0–3)	0.013^a^
Vomiting, frequency (IQR), times/day	1* (0–1)	1.5 (0–1)	0.9* (0–1)	1.8* (1–3)	0.030^b^
					0.015^c^
Fever, N (%)	1* (25)	11* (85)	5* (71)	15 (44)	0.022^d^
					0.035^e^
Fever duration (days)	1 (0–2)	2 (1–2)	1.4 (0–2)	1.1 (0–2)	
Viral shedding time (IQR), days	8.2* (5–13)	5.9* (2.6–7.1)	NA	NA	0.030^f^

IQR = interquartile range

^a^statistical comparison of immunocompromised NoV infections with immunocompetent non-NoV infections

^b^statistical comparison of immunocompetent non-NoV infections to immunocompromised NoV infections.

^c^statistical comparison of immunocompetent non-NoV infections to immunocompromised non-NoV infections.

^d^statistical comparison of immunocompromised NoV infections to immunocompetent NoV infections.

^e^statistical comparison of immunocompromised NoV infections to immunocompromised non-NoV infections.

^f^statistical comparison of immunocompromised NoV infections to immunocompetent NoV infections.

*statistical significance.

### Differences in the intestinal microbiota of immunodeficient and immunocompetent patients

**Fig 1:**
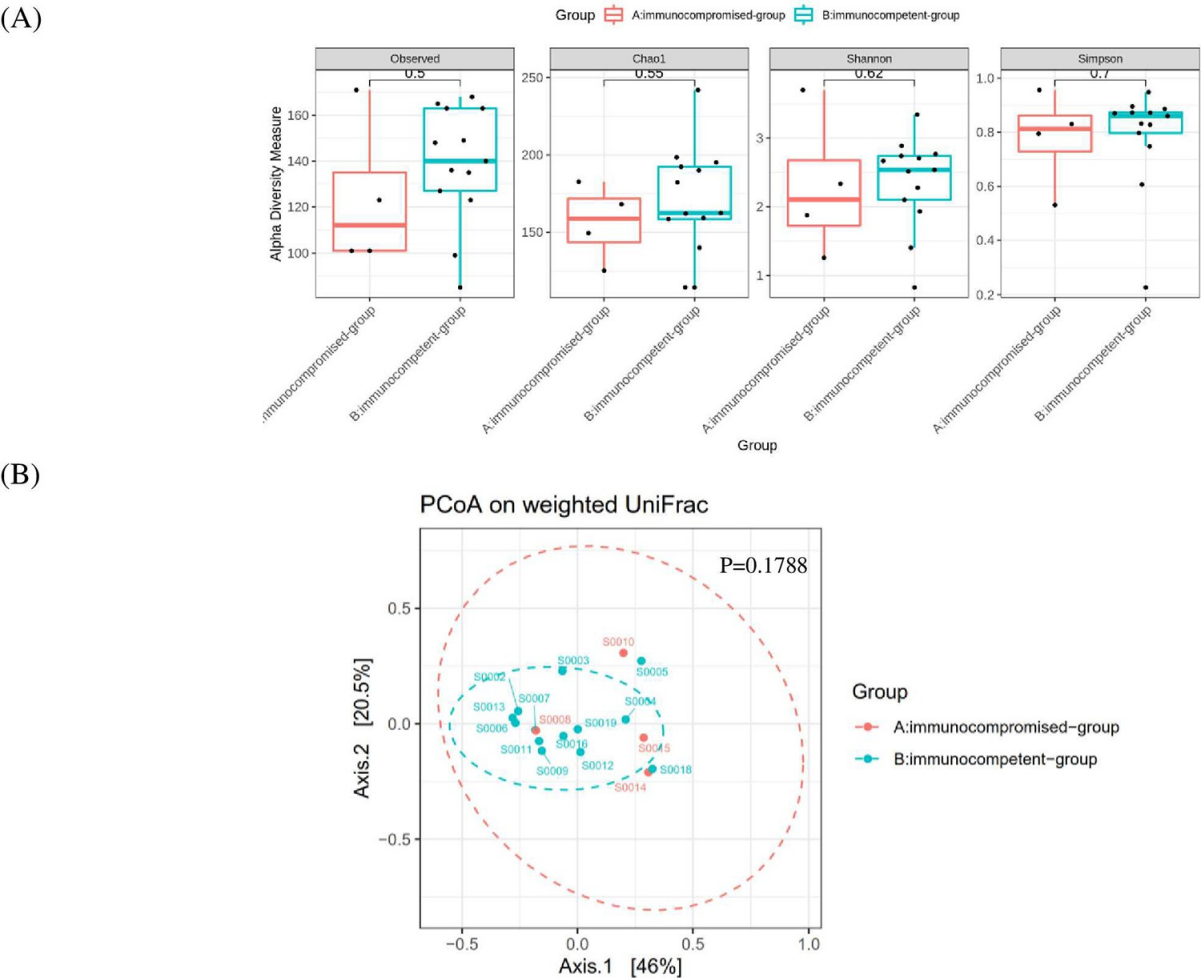
No significant difference in the community richness. (A) Alpha diversity between immunocompromised group and immunocompetent group with norovirus infection. (B) Beta diversity of bacterial communities between immunocompromised group and immunocompetent group with norovirus infection were calculated by the PCoA on weighted UniFrac.

**Fig 2:**
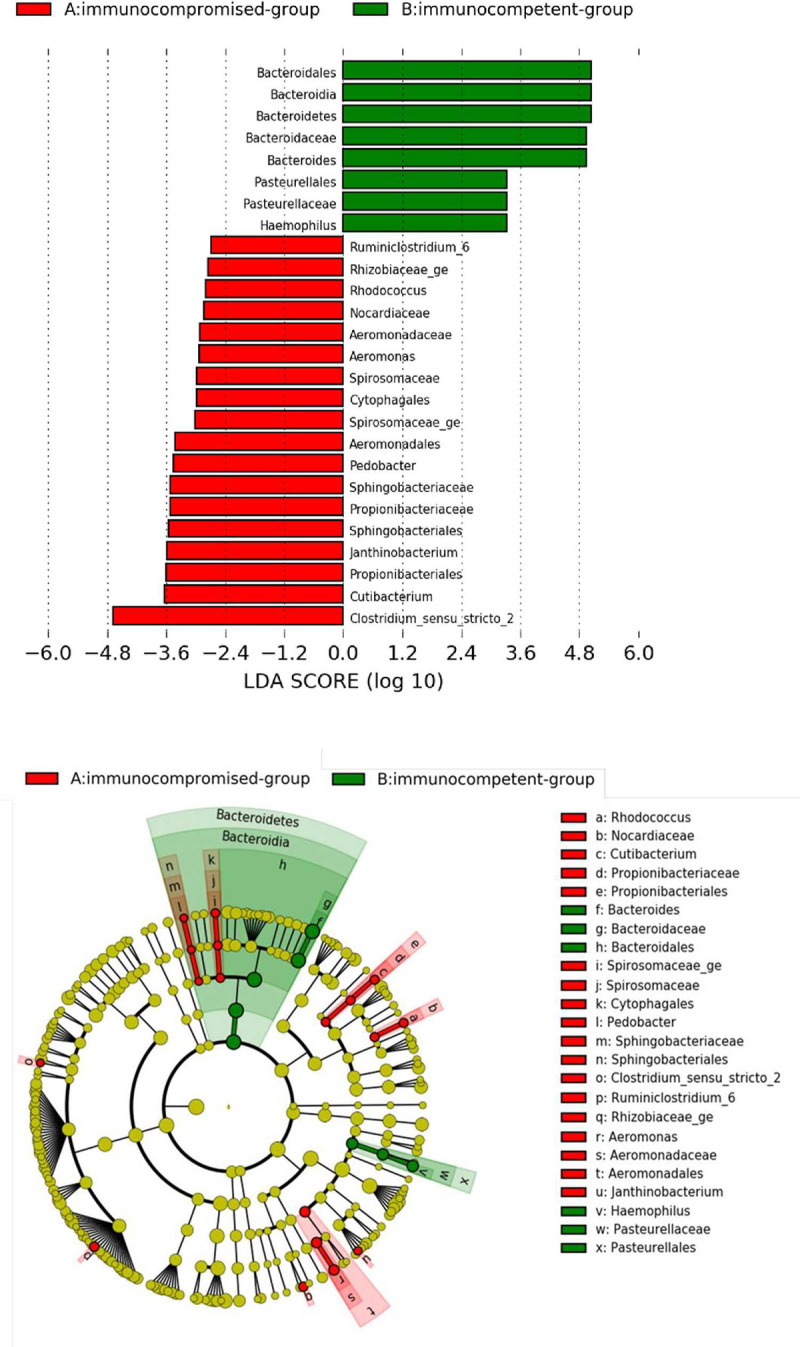
Gut microbiota is difference between immunocompromised group and immunocompetent group. Linear discriminant analysis (LDA) effect size (LEfSe) analysis of gut microbiota difference between immunocompromised group and immunocompetent group. Significant biomarkers were defined as taxa with a LDA score (log10) ≥ 2.

Enteric bacteria in the fecal samples of 17 norovirus AGE patients sent for intestinal microbiota metagenomics analysis by 16S rRNA sequencing were classified into 12 phyla, 18 classes, 74 families, and 211 genera. Table 2 shows the differences in the intestinal microbiota in norovirus gastroenteritis according to immune status. There was no significant difference in the community richness or alpha diversity between the immunocompromised and immunocompetent groups (Fig 1A). Weighted UniFrac PCoA analysis also showed no significant difference between the groups (Fig 1B). The LEfSe analysis shown a significant richness difference at the phylum level including Chloroflexi in immunocompromised group and Patescibacteria in immunocompetent groups (Fig 2). Also, significant microbial difference was found at the Family level including Corynebacteriaceae, Aeromonadaceae, Anaerolineaceae, Flexilinea, Sphingomonadaceae, Clostridia_Family_XI and Propionibacteriaceae with higher richness in immunocompromised group and Saccharimonadaceae in immunocompetent group. In Genus level, Finegoldia, Prevotella, Janthinobacterium, Aeromonas, Peptoniphilus, Scardovia, Turicibacter, Delftia, Ruminiclostridium_6, Sphingobium and Cutibacterium with higher richness in immunocompromised group and Saccharimonadaceae_ge and Oribacterium with higher richness in immunocompetent group (Fig 2).

**Table 2:** Different intestinal microbiota composition at level assignments in children with norovirus gastroenteritis by immunnity status.

Immunnity Status	Immunocompromised NoV infections (4)	Immunocompetent NoV infections (13)	*P* value
**Phylum level**			
*Chloroflexi*	0.027%±0.05%	0%	<0.001
*Patescibacteria*	0%	0.09%±0.06%	<0.001
**Family level**			
Corynebacteriaceae	0.598%±0.21%	0.033%±0.07%	0.015
*Aeromonadaceae*	0.104%±0.05%	0	<0.001
*Anaerolineaceae*	0.061%±0.04%	0	0.02
*Sphingomonadaceae*	0.024%±0.02%	0	0.02
*Clostridia_*Family_XI	0.192%±0.03%	0.03%±0.13%	0.033
*Propionibacteriaceae*	0.092%±0.13%	0	<0.001
*Saccharimonadaceae*	0	0.090%±0.06%	<0.001
**Genus level**			
*Finegoldia*	0.30%±0.25%	0.024%±0.013%	0.014
*Prevotella*	0.19%±0.12%	0.022%±0.015%	0.02
*Janthinobacterium*	0.048%±0.035%	0	<0.001
*Aeromonas*	0.067%±0.076%	0	<0.001
*Peptoniphilus*	0.10%±0.076%	0.016%±0.06%	0.015
*Scardovia*	0.10%±0.021%	0	<0.001
*Turicibacter*	0.06%±0.011%	0	<0.001
*Delftia*	0.06%±0.015%	0	<0.001
*Ruminiclostridium_6*	0.013%±0.006%	0	0.045
*Sphingobium*	0.024%±0.016%	0	0.02
*Cutibacterium*	0.035%±0.013%	0	0.015
*Saccharimonadaceae_ge*	0	0.187%±0.122%	0.033
Oribacterium	0	0.164%±0.074%	0.02

## Discussion

The NoV loads in the stools of cancer patients were calculated using real-time quantitative PCR (qPCR) to determine the genogroup-specific NoV load and examine the association with disease severity. One study of the association between the NoV stool load at the time of diagnosis and clinical outcome found that the NoV stool load was significantly associated with the severity of gastroenteritis, clinically significant dehydration, and intensive care unit admission; significantly higher viral loads at the time of diagnosis correlated with the clinical severity of both GI and GII NoV infections [21]. In our study, significantly more frequent diarrhea, lower fever prevalence, and longer virus shedding were found in NoV infections in immunocompromised patients compared to immunocompetent patients. We postulate that lower fever prevalence with a modest immune response to viral infection is associated with longer virus shedding in immunocompromised patients with NoVs infection.

Acute or persistent MNV infection was not associated with major disruptions of the microbial communities in Swiss Webster and C57BL/6 mice [22]. The abundance of bacterial groups such as *Faecalibacterium* and *Ruminococcus spp*., and lower IgA titers against NoV and rotavirus indicate links between host genetics, gut microbiota, and the susceptibility to viral infections in humans [23]. An animal study showed that MNV disrupts the epithelial barrier in animals, and is a potent colitogenic stimulus that largely depends on the presence of enteric microbiota. MNV may trigger overt clinical disease in individuals with a non-symptomatic predisposition to inflammatory bowel disease by impairing the intestinal mucosa [24]. Furthermore, a recent study revealed that naturally occurring strains isolated from human stool (*Klebsiella spp*., *Citrobacter spp*., *Bacillus spp*., *Enterococcus faecium*, and *Hafnia alvei*) and select reference strains (*Staphylococcus aureus* and *Enterobacter cloacae*) bound to representative human NoV strains (GII.4 New Orleans 2009 and Sydney 2012, GI.6) with high efficiency [25]. Thus, the dynamics between human NoVs and enteric bacteria have implications for NoV pathogenesis.

In the LEfSe analysis, a difference in *Saccharimonadaceae* was found in omeprazole-treated rats compared to untreated rats, implying a role of anti-acid mucosal immunity [26]. One microbiota profile study showed that *Oribacterium* was one of the major genera in early, invasive colorectal cancer [27]. We also found a difference in *Oribacterium* according to immune status, suggesting its role in the immunity of patients with malignancy other than colorectal cancer. *Finegoldia spp*. were one of three clinically relevant Gram-positive anaerobic cocci species with differences in susceptibility to different antibiotics [28]. We found *Finegoldia* was prevalent in immunocompromised patients and postulated that their overgrowth had a role in antibiotic susceptibility. Previously, we found no significant difference in flora diversity and taxonomic composition between normal children infected with norovirus and healthy children [29]. The current results also show that the microbiotas of children with low immune function are not significantly different from those of healthy children. Although there was no significant difference in the diversity of the intestinal microbiome in children infected with norovirus, the hierarchical classification showed a few specific differences in the distribution of the bacterial flora by genus and family. This may be related to the chemotherapy and other special therapies that such immunocompromised children undergo. Studies should examine the role and treatment of the intestinal microbiome in children with relatively low immune function during viral infections.

Our study is the first investigation of the clinical significance of norovirus gastroenteritis and the intestinal microbiota in immunocompromised children. This study had several limitations. First, a small number of patients were evaluated. Second, the patients took many different chemotherapy regimens before enrollment, which probably had undetermined effects on the intestinal microbiota. Third, the intestinal microbiota was investigated at a single time point, without longitudinal follow-up.

## Conclusions

In conclusion, we evaluated the clinical significance, virus shedding, and difference in the microbiotas of immunocompromised children with norovirus gastroenteritis. The results will facilitate studies of the interaction between the intestinal microbiota composition and norovirus infection in immunocompromised patients with the determination of bacterial infection control and probiotic supplements strategy to help prevention and eradication of severe infections in such patients.

## Supporting information

S1 Table (XLSX)
